# Association of Serum PSP/REG I*α* with Renal Function in Type 2 Diabetes Mellitus

**DOI:** 10.1155/2020/9787839

**Published:** 2020-03-23

**Authors:** Huimin Zhu, Xiangyun Zhu, Hao Lin, Dechen Liu, Yu Dai, Xianghui Su, Ling Li

**Affiliations:** ^1^Department of Endocrinology, Zhongda Hospital, School of Medicine, Southeast University, No. 87 Dingjiaqiao, Nanjing, Jiangsu 210009, China; ^2^Pancreatic Research Institute, Southeast University, China; ^3^Department of Clinical Science and Research, Zhongda Hospital, School of Medicine, Southeast University, No. 87 Dingjiaqiao, Nanjing, Jiangsu 210009, China; ^4^Nanjing Foreign Language School, Nanjing, Jiangsu 210009, China; ^5^Department of Endocrinology, Changji Branch, First Affiliated Hospital of Xinjiang Medical University, Xinjiang 831100, China

## Abstract

**Purpose:**

Pancreatic stone protein/regenerating protein I (PSP/REG I*α*) is a secretory protein mainly detected in the pancreas. Recent studies revealed increased serum PSP/REG I*α*) is a secretory protein mainly detected in the pancreas. Recent studies revealed increased serum PSP/REG I*α*) is a secretory protein mainly detected in the pancreas. Recent studies revealed increased serum PSP/REG I

**Methods:**

This cross-sectional study was conducted at Zhongda Hospital, affiliated with Southeast University in China. Serum PSP/REG I*α*) is a secretory protein mainly detected in the pancreas. Recent studies revealed increased serum PSP/REG I*α*) is a secretory protein mainly detected in the pancreas. Recent studies revealed increased serum PSP/REG I

**Results:**

Serum PSP/REG I*α*) is a secretory protein mainly detected in the pancreas. Recent studies revealed increased serum PSP/REG I*P* < 0.05). The level of PSP/REG I*α*) is a secretory protein mainly detected in the pancreas. Recent studies revealed increased serum PSP/REG I*α*) is a secretory protein mainly detected in the pancreas. Recent studies revealed increased serum PSP/REG I

**Conclusions:**

Serum PSP/REG I*α* level is significantly upregulated in T2DM patients and reflects renal function in both T2DM and nondiabetic control groups. The relationship between PSP/REG I*α* and eGFR suggested that PSP/REG I*α* might be a potential indicator of renal dysfunction.*α*) is a secretory protein mainly detected in the pancreas. Recent studies revealed increased serum PSP/REG I*α*) is a secretory protein mainly detected in the pancreas. Recent studies revealed increased serum PSP/REG I*α*) is a secretory protein mainly detected in the pancreas. Recent studies revealed increased serum PSP/REG I

## 1. Introduction

Type 2 diabetes mellitus (T2DM) is a metabolic disease that affects patients and relates with increased cancer incidence and poor prognosis [1, 2]. As a chronic disease, it is generally accepted that diabetes mellitus causes a variety of macrovascular and microvascular complications during the progression of the disease. Approximately 30-40% of diabetic patients develop nephropathy, and renal injury occurs in about a third of patients [3, 4]. Due to the growing incidence of T2DM, diabetic nephropathy has become the leading cause of end-stage renal disease (ESRD) worldwide. Accumulating evidence from experimental and clinical studies has demonstrated that renal inflammation plays a critical role in the development of diabetic nephropathy [5, 6]. Mou et al. reported that inflammatory stress may be caused by metabolic and hemodynamic disorders in diabetic nephropathy [7]. Inflammatory markers such as interleukin-1*β* and tumor necrosis factor-*α* upregulated in the patients with diabetic nephropathy [8].

Pancreatic stone protein/regenerating protein (PSP/REG I*α*) was originally a 16 kDa polypeptide found in pancreatic stones belonging to the superfamily of calcium-dependent lectin genes [9, 10]. It was initially discovered independently in the fields of pancreatitis, which is prominently upregulated when acute or chronic pancreatitis occurs [11]. It has subsequently been found to have a high degree of diagnostic accuracy in determining the seriousness of inflammation and predicting organ failure. In addition, PSP/REG I*α* has been demonstrated to increase *β* cell growth and regeneration by inducing cellular proliferation. PSP/REG I*α* messenger ribonucleic acid (mRNA) is mainly found in the pancreas, but its expression has also been detected in the gastric mucosa and the kidneys [9, 12]. It has been found in the urine and renal calculi of healthy individuals [13], which suggested a physiological role of PSP/REG I*α* in the kidney. Sobajima et al. reported that urinary PSP/REG I*α* was increased significantly in patients with various renal diseases, including diabetic nephropathy [14, 15]. Moreover, a previous study by the present researchers has found increased serum levels of PSP/REG I*α* in patients with diabetic nephropathy [16].

In this study, we measured serum PSP/REG I*α* levels in participants with and without diabetes to investigate whether PSP/REG I*α* was associated with renal function and further to evaluate its predictive value of kidney disease.

## 2. Methods

### 2.1. Study Subjects

Participants in this study were recruited from December 2018 to January 2019 in the Department of Endocrinology at Zhongda Hospital. The study was approved by the ethics committee of the hospital (2018ZDSYLL143-P01), and experimental methods were performed strictly in accordance with the approved guidelines. Informed consent was acquired from all participants. All patients in the T2DM group met the following inclusion criteria: a patient age > 10 years and a diagnosis of T2DM based on the 2012 criteria of the American Diabetes Association (ADA). Exclusion criteria were (1) enrolled in another trial, (2) pregnancy, (3) renal disease other than diabetic nephropathy, (4) acute complication of diabetes, (5) blood pressure ≥ 200/100 mmHg, (6) active infection, and (7) with tumor and take radiotherapy or chemotherapy within six months. 80 participants with T2DM and eGFR > 30 ml/min/1.73 m^2^ were randomly chosen and compared with an age-matched nondiabetic control group who underwent a regular health examination recruited from the hospital.

We collected demographic information including age, sex, height, weight, smoking status, and hypertension. From each patient, 5 ml of peripheral blood was collected and centrifuged directly for 6 min at a rotating speed of 3,000. The obtained serum was immediately frozen in sterile tubes at −80°C. Other clinical biochemical parameters, such as serum creatinine (SCr), blood urea nitrogen (BUN), uric acid (UA), total cholesterol (TC), and triglyceride (TG), were measured based on the standard methods. The center of Clinical Laboratory of Zhongda Hospital implements internal and external quality control procedures directed by a Chinese Quality Control Laboratory. Body mass index (BMI) was calculated using the following formula: BMI = body weight (kg)/body height (m^2^). The eGFR level was calculated using the modified Chronic Kidney Disease Epidemiology Collaboration (CKD-EPI) equation for Asians. The following formula was used: GFR (ml/min/1.73 m^2^) = 141 × min (SCr/0.7, 1)^−0.329^ × max (SCr/0.7, 1)^−1.209^ × 0.993^age^ × 1.018 (^∗^0.739 if female). Kidney function was classified using the method proposed by the U.S. National Kidney Foundation into three groups: normal (eGFR ≥ 90 ml/min/1.73 m^2^), mildly reduced (eGFR, 60 ml/min/1.73 m^2^ to 89 ml/min/1.73 m^2^), and moderately or severely reduced (eGFR < 60 ml/min/1.73 m^2^) [17, 18].

### 2.2. PSP/REG I*α* Enzyme-Linked Immunosorbent Assay (ELISA)

The enzyme-linked immunosorbent assay (ELISA) to measure human PSP/REG I*α* was performed as described previously [16], with guinea pig anti-human recombinant PSP/REG I*α* antibodies. The serum collected from the patients was prepared by centrifugation, and a sandwich method of ELISA was performed on 96-well plates. The plates were then blocked with 1% bovine serum albumin (BSA) for one hour. After that, guinea pig anti-PSP/REG I*α* antibody was coated on the bottom. The diluted recombinant human PSP/REG I*α* protein and serum were then used as supplements to the culture dish. After washing, rabbit anti-PSP/REG I*α* and then phosphatase-coupled rabbit anti-human PSP/REG I*α* were incubated. The reaction of the phosphatase with a substrate was determined at the absorbance of 405 nm on a microplate reader.

### 2.3. Statistical Analysis

Statistical analyses were conducted using SPSS 20.0 software. Descriptive analyses were presented as follows: (1) means ± SDs for normally distributed variables, (2) the medians (interquartile range (IQR)) for abnormally distributed variables, and (3) frequencies and percentages for count data. For the normally distributed variable, a *t*-test was performed to assess significant differences between the groups based on a test for homogeneity of variance. If the variable was nonnormally distributed, a Wilcoxon–Mann–Whitney test was used. A chi-squared test was performed for the count data to assess significant differences between the groups. The correlation between variables was presented using Spearman's rank correlation coefficient analyses. Ordinal logistic regression models were conducted in this study. As the dependent variable, eGFR was divided into three levels according to the National Kidney Foundation. All hypothesis tests used two-sided tests and set alpha at 0.05.

## 3. Results

### 3.1. Participants and Baseline Characteristics

A total of 183 subjects aged 14-82 participated in the study. The participants were divided into two groups, including 80 patients with T2DM and 103 subjects without T2DM enrolled as a control group. In the T2DM group, 7 patients were clinically diagnosed with diabetic nephropathy. The baseline characteristics of the participants are shown in Table 1. The proportion of males to females was significantly different between the T2DM and nondiabetic control group (*P* = 0.008). Among the two groups, no significant differences were observed in terms of age, BMI, TC, SCr, eGFR, and UA. The proportion of smoking was higher in the T2DM group (18.75%) than that in the nondiabetic control group (10.68%), but there was no significant difference in the value between the two groups (*P* = 0.121). The PSP/REG I*α* levels and the incidence of hypertension were significantly higher in individuals with T2DM compared to those in the control group (*P* = 0.025 and *P* < 0.001). Additionally, this is in accordance with our previous study showing that individuals with diabetic nephropathy had elevated PSP/REG I*α* levels.

### 3.2. The Correlation Analyses between Serum PSP/REG I*α* Levels and Renal Function

Considering that there may be some correlations between PSP/REG I*α* and renal function indicators, the researchers analyzed their correlations using Spearman's correlation coefficient analysis as the statistics in nonnormal distribution. It was observed that serum PSP/REG I*α* levels were negatively correlated with eGFR (*r* = −0.500, *P* < 0.001) and positively associated with age (*r* = 0.331, *P* < 0.001), SCr (*r* = 0.398, *P* < 0.001), and BUN (*r* = 0.351, *P* < 0.001).

To further investigate the correlations between PSP/REG I*α* and renal function indicators, analysis was performed on subjects according to whether they had T2DM. Spearman's correlation analysis indicated that serum PSP/REG I*α* levels were negatively correlated with eGFR (*r* = −0.519, *P* < 0.001) and positively associated with SCr (*r* = 0.440, *P* < 0.001), BUN (*r* = 0.348, *P* = 0.003), age (*r* = 0.259, *P* = 0.031), and UA (*r* = 0.314, *P* = 0.009) in patients with T2DM. Meanwhile, serum PSP/REG I*α* levels negatively correlated with eGFR (*r* = −0.474, *P* < 0.001) and associated significantly with age (*r* = 0.335, *P* = 0.001), serum Cr (*r* = 0.366, *P* < 0.001), and BUN (*r* = 0.346, *P* < 0.001) in subjects without T2DM (Table 2).

### 3.3. The Ordinal Multiple Logistic Regression Analysis Correlated with eGFR

In ordinal multiple logistic regression analysis, eGFRs were used as a grade-dependent variable in the model, which was classified into three levels by the National Kidney Foundation: normal (eGFR ≥ 90 ml/min/1.73 m^2^), mildly reduced (eGFR, 60 ml/min/1.73 m^2^ to 89 ml/min/1.73 m^2^), and moderately or severely reduced (eGFR < 60 ml/min/1.73 m^2^) [17, 18]. BUN, UA, hypertension, smoking, and PSP/REG I*α* levels were used as independent variables. The results illustrated that eGFRs showed association with PSP/REG I*α* levels in subjects of the nondiabetic control group (OR = 1.06, 95% CI: 1.04-1.09, *P* < 0.001) and the T2DM group (OR = 1.02, 95% CI: 1.01-1.03, *P* = 0.006) (Table 3).

## 4. Discussion

This study revealed PSP/REG I*α* levels in subjects with and without T2DM. We found that patients with T2DM had significantly elevated PSP/REG I*α* levels compared with those nondiabetic controls. In addition, we confirmed that PSP/REG I*α* levels were negatively correlated with eGFR and positively associated with age, SCr, and BUN in both groups. The ordinal multiple logistic regression analysis revealed substantially negative relationships between PSP/REG I*α* levels and eGFR.

First, it is noteworthy that PSP/REG I*α* is upregulated in T2DM patients. Initially, PSP and regenerating gene I*α* (REG I*α*) were found in the fields of pancreatitis and diabetes, respectively [10]. Sequence analysis later revealed that PSP and REG I*α* are indeed identical [11], and Graf et al. suggested that the combined term of PSP/REG I*α* could be used in the future. The regenerative capabilities of PSP/REG I*α* were identified in a screening study of genes related to beta cell regeneration firstly [19]. Subsequently, in diabetic rodent models, PSP/REG I*α* has been shown to increase the number of beta cells and stimulate beta cell proliferation under physiological conditions [9, 10]. Recently, strong evidence has shown that PSP/REG I*α* is associated with diabetes. Elevated PSP/REG I*α* levels have been observed in HNF1A–maturity onset diabetes of the young and the type 1 diabetes mellitus reported by Bacon et al. [20]. The present researchers have previously reported increased serum PSP/REG I*α* levels in T2DM patients, and these levels positively correlated with the duration of T2DM. With high levels of PSP/REG I*α*, the incidence of chronic complications is also increased [16]. In the present study, it was also confirmed that PSP/REG I*α* levels were higher in subjects in the T2DM group than those in the nondiabetic control group.

Another interesting observation in this study was that PSP/REG I*α* associated with eGFR and SCr. Previous studies reported that urinary PSP/REG I*α* excretion is increased in patients with renal disease and diabetic nephropathy [14, 21]. This study revealed that serum PSP/REG I*α* levels were associated with eGFR in patients with and without T2DM. There are three possible mechanisms to explain the correlation between PSP/REG I*α* and renal function. First, PSP/REG I*α* is mainly synthesized in the pancreas and as with other pancreatic enzymes, it can circulate in the blood. As a low-molecular-weight protein of 16 kDa, PSP/REG I*α* undergoes reabsorption in the proximal renal tubules [22, 23]. Given that PSP/REG I*α* is related to eGFR, the increased PSP/REG I*α* is more likely to reflect reduced glomerular filtration capacity rather than reabsorption from damaged renal tubules. In addition, PSP/REG I*α* may be participated in diabetic kidney hypertrophy as a kidney growth factor [24, 25]. As epidermal growth factor in the fluid of accumulating duct cysts has been shown to stimulate cyst growth, a similar role of PSP/REG I*α* in proximal tubule cysts is anticipated. Finally, many researchers have made suggestions that inflammation plays a crucial role in the development of diabetic nephropathy, and many studies have proved that higher levels of inflammatory biomarkers are associated with chronic kidney disease [26–29]. PSP/REG I*α* serves as an inflammatory factor that may be involved in renal disease.

The present researchers also found that smoking is a risk factor for the decline of eGFR in the nondiabetic control group, while it did not matter in the T2DM group. Researchers have reported that cigarette smoking has been identified as a modifiable risk factor for diseases because of its contrary effects. The amount of smoking and smoking habit may also have effects on the results; this is called a dose-response relationship [30]. In general conditions, T2DM patients with renal impairment could realize the damage caused by cigarettes. As a result, they may quit smoking so that the dose of smoking might be smaller than those in the nondiabetic control group. To sum up, more studies should certify the associations of smoking and eGFR in T2DM, and the mechanism needs to be further verified.

Circulating serum PSP/REG I*α* levels correlate with age, which implies that there is a positive correlation in the present study. This was identified by an age-dependent increase of PSP/REG I*α* levels in subjects in both groups. Schlapbach et al. [31] reported that age categories determined PSP/REG I*α* concentrations in healthy subjects. The lowest levels were seen in extremely preterm babies, while the highest levels were observed in children. This study provided normal values for specific ages that can be used to determine cutoff values for future PSP/REG I*α* level trials and demonstrated that PSP/REG I*α* increased from birth to childhood with an age development. However, the study did not clarify the relationship between age and PSP/REG I*α* levels in adults in a sickness state. Hence, further study is needed to confirm the relationships between age categories and PSP/REG I*α* levels in Asians.

To the present researchers' knowledge, this study is the first to recognize the correlations between serum PSP/REG I*α* and renal function in patients with and without T2DM. However, it also has some limitations. First, as a cross-sectional design, the sample size of this study is relatively small, so further prospective studies with larger samples are needed. Second, researchers need to detect PSP/REG I*α* levels in diabetic nephropathy even other kidney diseases to further ensure the value of PSP/REG I*α* in the diagnosis of renal function. Third, it is worth investigating further the associations between other diabetic complications, such as diabetic retinopathy, diabetic peripheral neuropathy, and diabetic foot.

## 5. Conclusions

This study provides evidence that PSP/REG I*α* is significantly upregulated in T2DM patients and reflects renal function in both T2DM and nondiabetic control subjects. Given the correlation between PSP/REG I*α* and eGFR, it is suggested that increased serum PSP/REG I*α* may reflect decreased glomerular filtration capacity. However, further research is needed to determine the value of PSP in the renal function of all the individuals and mechanisms involved.

## Figures and Tables

**Table 1:** Baseline characteristics of metabolic and laboratory parameters in patients.

	Control	T2DM	*χ* ^2^/*t*/*z*	*P*
	*N* = 103	*N* = 80		
Sex (male/female)	44/59	50/30	7.050	0.008
Age (years)	58.08 ± 14.29	61.58 ± 12.11	1.680	0.095
BMI (kg/m^2^)	23.64 ± 3.19	24.44 ± 3.12	1.617	0.108
Smoking (%)	11 (10.68)	15 (18.75)	2.406	0.121
Hypertension (%)	41 (39.81)	54 (67.5)	13.840	<0.001
TC (mmol/l)	4.59 ± 1.11	4.55 ± 1.30	-0.308	0.759
TG (mmol/l)	1.41 ± 0.91	2.19 ± 1.98	3.208	0.002
SCr (*μ*mol/l)	76 (50, 145)	75 (45, 137)	-0.502	0.615
eGFR (ml/min/1.73 m^2^)	88.93 ± 17.83	85.22 ± 26.58	-0.965	0.336
UA (*μ*mol/l)	320.90 ± 78.02	309.58 ± 86.43	-1.090	0.277
BUN (mmol/l)	5.24 ± 1.53	6.26 ± 3.74	2.209	0.029
PSP/REG I*α* (ng/ml)	36.81 (13, 140.93)	47.01 (13, 694)	-2.248	0.025

T2DM = type 2 diabetes mellitus; control = without diabetes mellitus. Data are presented as *n* (%), mean ± SD, or median (interquartile range) as appropriate. BMI = body mass index; TC = total cholesterol; TG = triglyceride; SCr = serum creatinine; eGFR = estimated glomerular filtrations rate; UA = uric acid; BUN = blood urea nitrogen. Significance: *P* < 0.05 compared with control.

**Table 2:** Relationship of metabolic and laboratory parameters with PSP/REG I*α*.

	Control (*N* = 103)		T2DM (*N* = 80)	
	*r*	*P*	*r*	*P*
Age (years)	0.335	0.001	0.259	0.031
BMI (kg/m^2^)	0.074	0.457	-0.041	0.739
BUN (mmol/l)	0.346	<0.001	0.348	0.003
SCr (*μ*mol/l)	0.366	<0.001	0.440	<0.001
eGFR (ml/min/1.73 m^2^)	-0.474	<0.001	-0.519	<0.001
UA (*μ*mol/l)	0.106	0.287	0.314	0.009
TC (mmol/l)	-0.104	0.308	0.001	0.993
TG (mmol/l)	-0.088	0.389	0.093	0.448

Significance: *P* < 0.05. Spearman's rank correlation coefficient: *r*. BMI = body mass index; BUN = blood urea nitrogen; SCr = serum creatinine; eGFR = estimated glomerular filtrations rate; UA = uric acid; TC = total cholesterol; TG = triglyceride.

**Table 3:** Ordinal multiple logistic regression showing variables independently associated with serum eGFR levels.

Variables	Control (*N* = 103)			T2DM (*N* = 80)		
	*β*	OR (95% CI)	*P*	*β*	OR (95% CI)	*P*
BUN	0.134	1.14 (0.85-1.54)	0.376	0.497	1.64 (1.16-2.30)	0.004
UA	0.004	1.00 (0.99-1.01)	0.215	0.003	1.00 (0.99-1.01)	0.413
Hypertension	0.483	1.62 (0.64-4.08)	0.304	1.227	3.41 (1.04-11.57)	0.049
Smoking	1.800	5.33 (1.23-27.54)	0.024	-1.103	0.33 (0.08-1.42)	0.137
PSP/REG I*α*	0.061	1.06 (1.04-1.09)	<0.001	0.020	1.02 (1.01-1.03)	0.006

CI = confidence interval; OR = odds ratio; significance: *P* < 0.05; BUN = blood urea nitrogen; UA = uric acid.

## Data Availability

The datasets generated and/or analyzed during this study are not publicly available, owing to currently ongoing research studies, but the data are available from the corresponding author on reasonable request.

## Abbreviations

PSP/REG I*α*: Pancreatic stone protein/regenerating protein I*α*
T2DM: Type 2 diabetes mellitus
BUN: Blood urea nitrogen
Scr: Serum creatinine
UA: Uric acid
eGFR: The estimated glomerular filtration rate
ESRD: End-stage renal disease
mRNA: Messenger ribonucleic acid
ADA: American Diabetes Association
BMI: Body mass index
CKD-EPI: Chronic Kidney Disease Epidemiology Collaboration
ELISA: Enzyme-linked immunosorbent assay
BSA: Bovine serum albumin
CI: Confidence interval
OR: Odds ratio.
